# A Two-Photon Fluorescent Probe for the Visual Detection of Peroxynitrite in Living Cells and Zebrafish

**DOI:** 10.3390/molecules27154858

**Published:** 2022-07-29

**Authors:** Zhencai Xu, Jin Qian, Yufeng Ge, Yalan Wang, Hongwei Chen

**Affiliations:** 1 Guanyun People’s Hospital, Lianyungang 222000, China; zhaoll0326@163.com; 2 Modern Education Technology Center, Department of Critical Care Medicine, The First Affiliated Hospital of Hainan Medical University, Hainan Medical University, Haikou 571199, China; jang9116@hainmc.edu.cn; 3 Department of Emergent Trauma Surgery, Qilu Hospital of Shandong University, Jinan 250012, China; geyufengqilu@163.com

**Keywords:** peroxynitrite, fluorescent probe, cell imaging, zebrafish

## Abstract

Peroxynitrite (ONOO^−^), as an important reactive oxygen species (ROS), holds great potential to react with a variety of biologically active substances, leading to the occurrence of various diseases such as cancer and neurodegenerative diseases. In this work, we developed a novel mitochondria-localized fluorescent probe, HDBT-ONOO^−^, which was designed as a mitochondria-targeting two-photon fluorescence probe based on 1,8-naphthylimide fluorophore and the reactive group of 4-(bromomethyl)-benzene boronic acid pinacol ester. More importantly, the probe exhibited good biocompatibility, sensitivity, and selectivity, enabling its successful application in imaging the generation of intracellular and extracellular ONOO^−^. Furthermore, exogenous and endogenous ONOO^−^ products in live zebrafish were visualized. It is greatly expected that the designed probe can serve as a useful imaging tool for clarifying the distribution and pathophysiological functions of ONOO^−^ in cells and zebrafish.

## 1. Introduction

Peroxynitrite (ONOO^−^), as representative active oxygen, shows high reactivity in living organisms [1,2,3]. It plays an important role in the physiological and pathological processes of living systems [4,5,6]. The transformation of ONOO^−^ in biological systems mainly involves two pathways: protonation to generate peroxynitrous acid (ONOOH), and quickly reacting with CO_2_ to form a short-lived intermediate nitrosoperoxycarbonate (ONOOCO_2_^−^) [7,8]. ONOO^−^ can diffuse freely through the phospholipid membrane bilayer, and its metabolites can react with a variety of important biomolecules (including proteins, lipids, and nucleic acids), eventually leading to mitochondrial dysfunction and cell death [9,10,11]. However, the overproduction of ONOO^−^ in vivo can lead to abnormalities in a variety of life targets, such as DNA, proteins, enzymes, and nucleic acids, which can, in turn, cause many diseases, such as cancer, Alzheimer’s disease, and nervous system degeneration [12,13,14,15]. Therefore, it is urgently needed to develop an accurate ONOO^−^ detection method that plays an important role in the indepth understanding of complex diseases in living systems.

Traditional methods of detecting ONOO^−^ are usually time-consuming and expensive [16,17,18]. ONOO^−^ also has some features such as instantaneity, low lifetime, low in vivo concentration that render its effective capture and further detection great challenges. Fluorescence imaging technology has developed rapidly in recent years. It possesses the advantages of high sensitivity, selectivity, in situ detection, and noninvasiveness, which have attracted the attention of an increasing number of researchers [19,20,21,22,23,24]. A variety of fluorescent probes for detecting ONOO^−^ have been developed and have had widespread applications [22,23,25,26,27,28], but there are few two-photon probes used to detect the distribution level of ONOO^−^ in subcellular organelles. Two-photon imaging itself has the characteristics of high resolution, high throughput, noninvasiveness, and excellent imaging depth [29,30,31,32], which enables it to exert the dynamical visualization functions of living organisms and cells in an active state, thereby facilitating researchers in exploring the changing levels of ONOO^−^ in cells and organisms in physiological and stimulating states.

On the basis of the above information, we developed a novel two-photon fluorescent probe, HDBT-ONOO^−^, for monitoring ONOO^−^ in mitochondria. HDBT-ONOO^−^ was equipped with cationic triphenylphosphine and borates groups as mitochondrial targeting groups and peroxynitrite responsive sites, respectively. Spectroscopic experiments confirmed the excellent selectivity and sensitivity of HDBT-ONOO^−^, and cell imaging experiments demonstrated that HDBT-ONOO^−^ showed mitochondria-targeting abilities and could be successfully used for two-photon imaging of ONOO^−^ variations in mitochondria in living cells. The following zebrafish experiments further proved that HDBT-ONOO^−^ had excellent sensitivity in the detection of the level of ONOO^−^ in the body. The above results confirm that HDBT-ONOO^−^ holds great application value, and lay the foundation for us to further study the pathophysiological processes with ONOO^−^ involved in vivo.

## 2. Experimental Section

### 2.1. General Comments

Details of materials and measurements were transferred to Supplementary Materials.

### 2.2. Synthesis of HDBT-ONOO^−^

Intermediate compounds in Supporting Information (Figure S1).

A mixture of compound 4 (258 mg, 0.5 mmol), 4-(bromomethyl)-benzene boronic acid pinacol ester (297 mg, 1 mmol) and anhydrous K_2_CO_3_ (138 mg, 1 mmol) in 10 mL of DMF was refluxed at 80 °C overnight. The solvent was removed, and the obtained crude product was purified with column chromatography using dichloromethane:methanol (50:1–20:1) to obtain a yellow solid HDBT-ONOO^−^. ^1^H NMR (500 MHz, CDCl_3_) δ 8.66–8.49 (m, 2H), 8.17 (s, 1H), 7.87 (dd, *J* = 33.0, 7.6 Hz, 3H), 7.73 (d, *J* = 10.4 Hz, 6H), 7.47 (ddd, *J* = 39.3, 26.7, 7.6 Hz, 11H), 7.07 (dd, *J* = 26.9, 8.1 Hz, 1H), 5.24 (s, 2H), 4.29 (t, *J* = 6.4 Hz, 2H), 2.42 (s, 2H), 2.08 (s, 2H), 1.37 (s, 12H). ^13^C NMR (126 MHz, CDCl_3_) δ 164.4, 160.7, 138.5, 135.3, 135.2, 135.1, 133.5, 131.7, 130.8, 130.8, 128.8, 128.7, 128.6, 127.4, 126.7, 126.1, 115.1, 106.5, 84.0, 83.9, 70.84 (s), 65.5 (s), 27.9 (s), 24.9. LC-HRMS (ESI, negative ion mode): *m*/*z* [C_32_H_22_N_3_O_5_P^+^], calcd,732.3049; found [M]: 732.3047.

### 2.3. Statistical Methods

The experimental data were analyzed using SPSS17. The 0 software package was used for statistical processing, measurement data are expressed as mean ± SD, and the *t*-test of two independent samples was used for comparison between groups. *p* < 0.05 was considered to be statistically significant.

## 3. Results and Discussion

### 3.1. Design of the Probe HDBT-ONOO^−^

**Scheme 1:**
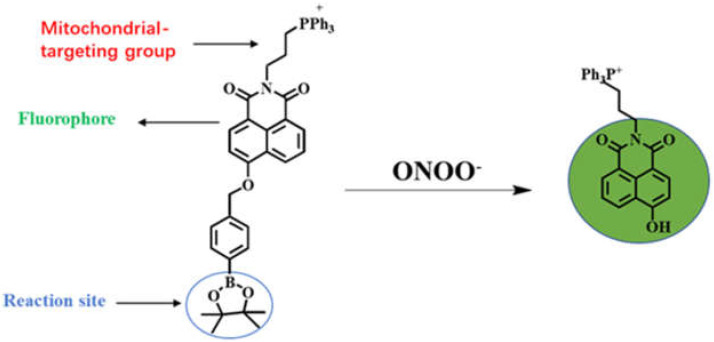
Composition of HDBT-ONOO^−^ and its reaction with ONOO^−^.

The structure of HDBT-ONOO^−^ to ONOO^−^ and the proposed response mechanism are presented in Scheme 1. As shown in Scheme 1, the probe was designed by binding a 1,8-naphthylimide fluorophore scaffold modified with 4-bromomethylphenylboronic acid pinacol ester, where it exhibited bright fluorescence upon the conversion of borates into the corresponding phenol by ONOO^−^. The introduction of cationic triphenylphosphine enabled the probe to be highly localized near the subcellular organelle mitochondria [33,34,35]. The probe was employed for the sensitive and selective detection of both exogenous and endogenous ONOO^−^. The structural characterization of target substances was performed with ^1^H NMR, ^13^C NMR, and HR–MS (Figures S5–S11).

### 3.2. Spectral Response of HDBT-ONOO^−^ to ONOO^−^

**Figure 1:**
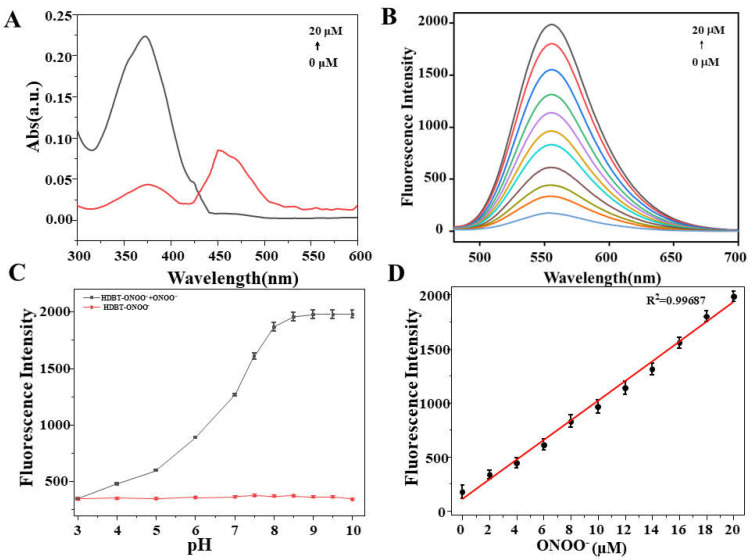
(**A**) UV–vis absorption spectra of HDBT-ONOO^−^ (10 μM) after adding various concentrations of ONOO^−^ (0–20 μM); (**B**) fluorescence response of HDBT-ONOO^−^ (10 μM) to different concentrations of ONOO^−^ (0–20 μM); (**C**) fluorescence intensities of HDBT-ONOO^−^ (10 μM) after adding ONOO^−^ (20 μM) under different pH conditions; (**D**) linear relationship between HDBT-ONOO^−^ (10 μM) and ONOO^−^ concentration (0–20 μM). Experiments were repeated 3 times, and data are shown as the mean (± SD). The spectrum was obtained in PBS solution containing 5% DMSO (10 mM, pH 7.4, λ_ex_ = 450 nm) at room temperature.

**Figure 2:**
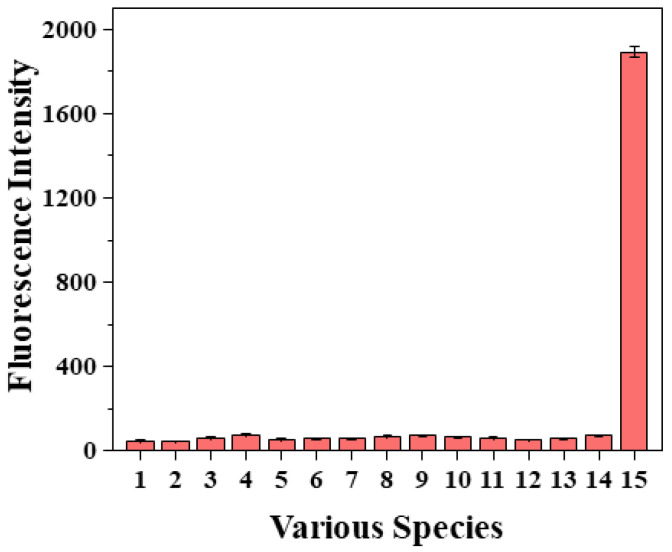
The response of HDBT-ONOO^−^ (10 μM) to various species: (1) probe only; (2) ClO^−^; (3) NO; (4) ^•^OH; (5) ^•^O_2_–; (6) ^1^O_2_; (7) H_2_O_2_; (8) Na^+^; (9) Ca^2+^; (10) Mg^2+^; (11) Zn^2+^; (12) Fe^2+^; (13) Al^3+^; (14) Cu^2+^; (15) ONOO^−^. The experiments were repeated three times, and the data are shown as the mean (± SD). All data were obtained in PBS (10 mM, pH 7.4) at room temperature. λ_ex_ = 450 nm.

We explored the spectral properties of HDBT-ONOO^−^. The absorption and fluorescence emission spectra of the probes were first evaluated separately under simulated physiological conditions. As shown in Figure 1A, the addition of ONOO^−^ resulted in the gradual disappearance of the absorption band centered at 372 nm in HDBT-ONOO^−^, while a new red-shifted band appeared at around 450 nm. Next, we investigated the fluorescence spectra of HDBT-ONOO^−^. As shown in Figure 1B, the fluorescence intensity of the probe at 558 nm increased as ONOO^−^ concentration increased. In addition, we found a satisfactory linear response relationship between the fluorescence intensities of HDBT-ONOO^−^ and the concentrations of ONOO^−^ (Figure 1D). The linear fitting equation was F_558nm_ = 91.18243 [ONOO^−^] + 107.1069, and the correlation coefficient (R^2^) was 0.99687. On the basis of the standard method of 3σ/k, the detection limit of ONOO^−^ was calculated to be 56 nM. Later, we investigated whether pH could affect changes in probe fluorescence intensity. As shown in Figure 1C, HDBT-ONOO^−^ showed a weak fluorescence signal in the studied pH range (3.0–10.0). After adding a certain amount of ONOO^−^ (20 μM), the fluorescence intensity of the probe gradually increased with the increase in pH, and the fluorescence remained relatively stable in the pH range of 7–10. Subsequently, the fluorescence intensity gradually weakened with the further increase in alkalinity. These results indicate that HDBT-ONOO^−^ could be suitable for the detection of ONOO^−^ content under physiological conditions. The time course of HDBT-ONOO^−^ fluorescence emission at 558 nm after the addition of ONOO^−^ (20 μM) was next investigated. Figure S2 shows that the fluorescence intensity of the probe increased with time and reached a maximum at around 30 s. To further confirm the specificity of the probe HDBT-ONOO^−^ for ONOO^−^, we tested the ability of the probe HDBT-ONOO^−^ to discriminate ONOO^−^ from other biologically relevant species, including metal cations (Na^+^, Ca^2+^, Mg^2+^, Zn^2+^, Fe^2+^, Al^3+^, Cu^2+^) and other ROS. As shown in Figure 2, only ONOO^−^ caused an observable fluorescence response, which indicated that HDBT-ONOO^−^ had excellent selectivity and selectivity to ONOO^−^ (Figure 2). These results indicate that HDBT-ONOO^−^ could be suitable for detecting ONOO^−^ content under physiological conditions.

### 3.3. Fluorescence Imaging in Living Cells

**Figure 3:**
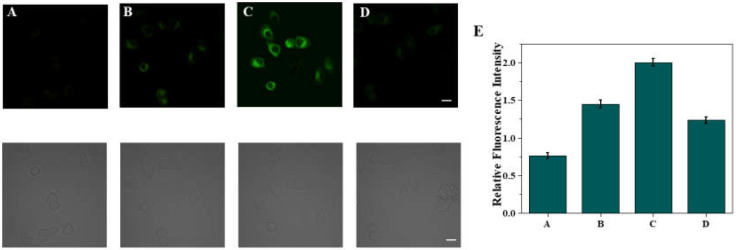
Confocal fluorescence imaging of HDBT-ONOO^−^ with exogenous addition of ONOO^−^ donor (SIN-1) in HeLa cells. HeLa cells were incubated with HDBT-ONOO^−^ (10 µM) and (**A**) 0, (**B**) 0.5, (**C**) 1.2 mM SIN-1, and (**D**) 200 μM ebselen, and 1.2 mM SIN-1. (**E**) Relative fluorescence intensity of (**A**–**D**). Cells were stained with HDBT-ONOO^−^ (10 µM) for 30 min, washed with PBS, and imaged by confocal microscopy. λ_ex_ = 880 nm, scale bar: 30 µm. Error bars represent the standard deviations of three separate measurements (n = 3).

Encouraged by the above experiments, we then explored the potential of the probe in biological applications. As shown in Figure S3, HeLa, RAW 264.7, and HepG 2 cells maintained a high survival rate after being exposed to probe concentrations below 70 μM. SIN-1 is a well-known donor of ONOO^−^ [36]. As shown in Figure 3, as the concentration of SIN-1 increased, the fluorescence intensity of the probe gradually increased and reached a maximum with the concentration of SIN-1 at 1.2 m. Figure 3D shows that the fluorescence intensity of HDBT-ONOO^−^ was significantly attenuated after the addition of ONOO^−^ scavenger ebselen (200 µM). This revealed that our probe HDBT-ONOO^−^ could sensitively detect the changes in exogenous ONOO^−^ in cells.

**Figure 4:**
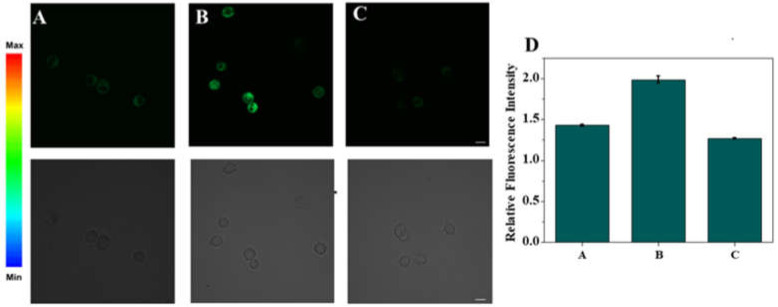
Fluorescence imaging of ONOO^−^ in RAW 264.7 cells treated with (**A**) probe only, (**B**) 1.2 μg mL^−1^ LPS 16 h, 70 ng mL^−1^ IFN-γ 4 h, and (**C**) LPS, IFN-γ + 150 μM ebselen. (**D**) Relative fluorescence intensity of (**A**–**C**). Cells were stained with HDBT-ONOO^−^ (10 µM) for 30 min, washed with PBS, and imaged by confocal microscopy. λ_ex_ = 880 nm, scale bar: 30 µm. Error bars represent the standard deviations of three separate measurements (n = 3).

To verify the reactivity of the probe to the intracellular ONOO^−^, the fluorescence imaging of RAW 264.7 cells was performed. Lipopolysaccharide (LPS) and interferon-γ (IFN-γ) could stimulate the production of ROS/RNS in RAW 264.7 cells to produce endogenous ONOO^−^ [37,38,39,40]. As shown in Figure 4, significant green fluorescence in the cytoplasm indicated that HDBT-ONOO^−^ could respond with ONOO^−^ in the cell. Then, ebselen was added in the presence of LPS and IFN-γ, and as expected, no changes in fluorescence were observed (Figure 4C). These results indicate that HDBT-ONOO^−^ can detect endogenous ONOO^−^ in living cells, so it has potential for imaging applications.

**Figure 5:**
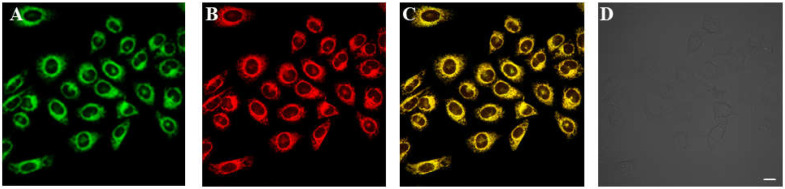
Fluorescence images of mitochondrial ONOO^−^ in HepG2 cells. Cells were stained with 10 µM HDBT-ONOO^−^ or costained with 50 nM MT Deep Red for 60 min, and then stimulated with 1.2 mM SIN-1 for another 90 min before imaging. (**A**) Probe-stained HepG 2 cells were stimulated with 1.2 mM SIN-1. (**B**) Costaining and imaged with MT Deep Red. (**C**) Merged images of (**A**,**B**). (**D**) Bright field of (**A**). Images were acquired using a fluorescent microscope (green channel with 458 nm excitation and 510–590 nm collection, a red channel with 633 nm excitation and 650–700 nm collection). Scale bar: 30 µm.

**Figure 6:**
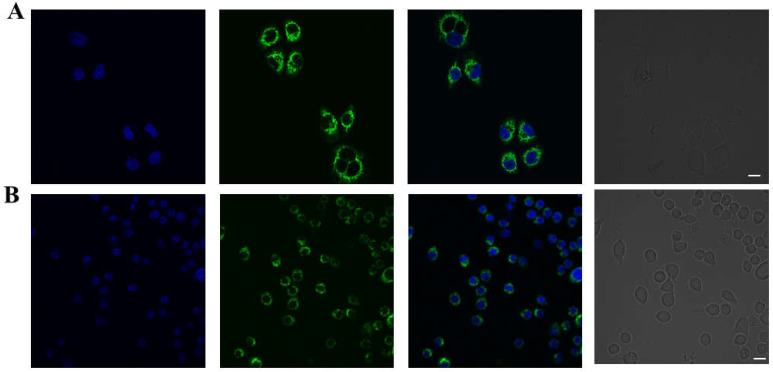
Fluorescence image of ONOO^−^ in the cytoplasm of HepG2 cells. Cells were stained with 10 µM HDBT-ONOO^−^ or costained with 10 µM DAPI for 60 min, and then stimulated with 1.2 mM SIN-1 for another 90 min before imaging. (**A**) (left to right) DAPI in HeLa cells, probe + SIN-1, DAPI + probe +S IN-1, bright-field image of (**A**); (**B**) (left to right) DAPI in RAW 264.7 cells, probe + SIN-1, DAPI + probe + SIN-1, bright-field image of (**B**). The images were acquired using a fluorescent microscope (green channel with 458 nm excitation and 520–590 nm collection, a blue channel with 405 nm excitation and 430–480 nm collection). Scale bar: 30 µm.

Lastly, using Mito-Tracker Deep Red (MT Deep Red), a commercially available mitochondrial dye, the subcellular distribution map of HDBT-ONOO^−^ was drawn through costaining experiments. As shown in Figure 5, the green signal generated by HDBT-ONOO^−^ in response to exogenous ONOO^−^ overlapped well with the red fluorescence of MT Deep Red. The calculated Pearson correlation coefficients were evaluated as 0.88, providing direct evidence that the probe could be selectively and effectively localized to the mitochondria of HepG 2 cells to detect slight ONOO^−^ level changes. To study the distribution of the probe in the cell, HDBT-ONOO^−^ was incubated with the ONOO donor SIN-1 or nuclear dye DAPI (4′,6-diamidino-2-phenylindole, which was a fluorescent dye that can bind strongly to DNA). As shown in Figure 6, in RAW 264.7 and HeLa cells, the fluorescence of the probe was mainly distributed in the cytoplasm, not in the nucleus.

### 3.4. Imaging of ONOO^−^ in Zebrafish

**Figure 7:**
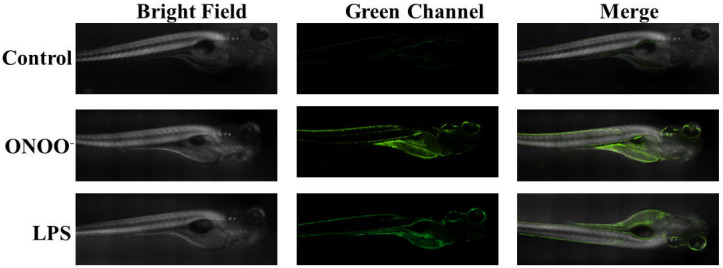
Bright-field and fluorescence imaging of exogenous and endogenous ONOO^−^ generation in zebrafish by using HDBT-ONOO^−^ as the probe. λ_ex_ = 450 nm.

Using HDBT-ONOO^−^ as a probe, we demonstrated the fluorescence imaging of exogenous and endogenous ONOO^−^ generation in a zebrafish model. As shown in Figure 7, we observed only a weak fluorescence response in the control group. After the incubation of zebrafish with ONOO^−^, bright green fluorescence was observed. Similarly, upon stimulation by LPS, endogenous zebrafish ONOO^−^ was formed and thus exhibited intense fluorescence. These images demonstrate that HDBT-ONOO^−^ can sensitively image both exogenous and exogenous ONOO^−^ production in zebrafish.

## 4. Conclusions

In conclusion, we developed a novel two-photon fluorescent probe, HDBT-ONOO^−^, for sensitive detection of mitochondrial ONOO^−^ in living cells. It consisted of 1,8-naphthalimide fluorophore modified with a triphenylphosphonium targeting group and a boronate-based molecule switch. The probe demonstrated the desired properties of high selectivity, excellent water solubility, and physiological pH response, along with low cytotoxicity, enabling the tracking of mitochondrial ONOO^−^ in living cells. Thus, the probe might serve as a tool for probing the biological roles of mitochondrial ONOO^−^ and facilitating the mechanistic investigation of mitochondria-targeting anticancer agents. Lastly, zebrafish experiments implied the potential application of HDBT-ONOO^−^ in the studies of the ONOO^−^ roles in live organisms. Therefore, the probe may be promising as a tool for exploring the biological role of mitochondrial ONOO^−^, and to promote a deep understanding of the molecular events and mechanism of ONOO^−^ in the body.

## Data Availability

The data that support the findings of this study are available from the corresponding author upon reasonable request.

## Supplementary Materials

The following supporting information can be downloaded at: https://www.mdpi.com/article/10.3390/molecules27154858/s1, Figure S1: Synthesis of HDBT-ONOO^−^; Figure S2: Temporal response kinetics of HDBT-ONOO^−^ and ONOO^−^; Figure S3: Cytotoxic assay; Figure S5: ^1^H NMR of compound 3; Figure S6: ^13^C NMR of compound 3; Figure S7: ^1^H NMR of compound 4; Figure S8: ^13^C NMR of compound 4; Figure S9: ^1^H NMR of HDBT-ONOO^−^; Figure S10: ^13^C NMR of HDBT-ONOO^–;^ Figure S11:HR-MS of HDBT-ONOO^−^ [41,42,43,44,45,46,47,48].

## Sample Availability

Samples of the compounds HDBT-ONOO^−^ are available from the authors.

## References

[1] Serrano-Luginbuehl S., Kissner R., Koppenol W.H. (2018). Reaction of CO_2_ with ONOO^−^: One Molecule of CO_2_ Is Not Enough. Chem. Res. Toxicol..

[2] Beckman J.S., Beckman T.W., Chen J., Marshall P.A., Freeman B.A. (1990). Apparent hydroxyl radical production by peroxynitrite: Implications for endothelial injury from nitric oxide and superoxide. Proc. Natl. Acad. Sci. USA.

[3] Ischiropoulos H., Zhu L., Chen J., Tsai M., Martin J.C., Smith C.D., Beckman J.S. (1992). Peroxynitrite-mediated tyrosine nitration catalyzed by superoxide dismutase. Arch. Biochem. Biophys..

[4] Ferrer-Sueta G., Radi R. (2009). Chemical biology of peroxynitrite: Kinetics, diffusion, and radicals. ACS Chem. Biol..

[5] Koppenol W.H., Moreno J.J., Pryor W.A., Ischiropoulos H., Beckman J.S. (1992). Peroxynitrite, a cloaked oxidant formed by nitric oxide and superoxide. Chem. Res. Toxicol..

[6] Nathan C., Cunningham-Bussel A. (2013). Beyond oxidative stress: An immunologist’s guide to reactive oxygen species. Nat. Rev. Immunol..

[7] Radi R. (2013). Peroxynitrite, a stealthy biological oxidant. J. Biol. Chem..

[8] Szabo C. (2003). Multiple pathways of peroxynitrite cytotoxicity. Toxicol. Lett..

[9] Alvarez B., Radi R. (2003). Peroxynitrite reactivity with amino acids and proteins. Amino Acids.

[10] Ferrer-Sueta G., Campolo N., Trujillo M., Bartesaghi S., Carballal S., Romero N., Alvarez B., Radi R. (2018). Biochemistry of Peroxynitrite and Protein Tyrosine Nitration. Chem. Rev..

[11] Szabo C., Ischiropoulos H., Radi R. (2007). Peroxynitrite: Biochemistry, pathophysiology and development of therapeutics. Nat. Rev. Drug Discov..

[12] Pacher P., Beckman J.S., Liaudet L. (2007). Nitric oxide and peroxynitrite in health and disease. Physiol. Rev..

[13] Torreilles F., Salman-Tabcheh S., Guerin M., Torreilles J. (1999). Neurodegenerative disorders: The role of peroxynitrite. Brain Res. Rev..

[14] Szabo C. (2009). Role of nitrosative stress in the pathogenesis of diabetic vascular dysfunction. Br. J. Pharmacol..

[15] Okun Z., Kupershmidt L., Amit T., Mandel S., Bar-Am O., Youdim M.B., Gross Z. (2009). Manganese corroles prevent intracellular nitration and subsequent death of insulin-producing cells. ACS Chem. Biol..

[16] Daiber A., Oelze M., August M., Wendt M., Sydow K., Wieboldt H., Kleschyov A.L., Munzel T. (2004). Detection of superoxide and peroxynitrite in model systems and mitochondria by the luminol analogue L-012. Free Radic Res..

[17] Dikalov S., Grigor’ev I.A., Voinov M., Bassenge E. (1998). Detection of superoxide radicals and peroxynitrite by 1-hydroxy-4-phosphonooxy-2,2,6,6-tetramethylpiperidine: Quantification of extracellular superoxide radicals formation. Biochem. Biophys. Res. Commun..

[18] Xue J., Ying X., Chen J., Xian Y., Jin L. (2000). Amperometric ultramicrosensors for peroxynitrite detection and its application toward single myocardial cells. Anal. Chem..

[19] Yamada K., Mito F., Matsuoka Y., Ide S., Shikimachi K., Fujiki A., Kusakabe D., Ishida Y., Enoki M., Tada A. (2016). Fluorescence probes to detect lipid-derived radicals. Nat. Chem. Biol..

[20] Kawatani M., Yamamoto K., Yamada D., Kamiya M., Miyakawa J., Miyama Y., Kojima R., Morikawa T., Kume H., Urano Y. (2019). Fluorescence Detection of Prostate Cancer by an Activatable Fluorescence Probe for PSMA Carboxypeptidase Activity. J. Am. Chem. Soc..

[21] Ning J., Tian Z., Wang J., Wang B., Tian X., Yu Z., Huo X., Feng L., Cui J., James T.D. (2022). Rational Design of a Two-Photon Fluorescent Probe for Human Cytochrome P450 3A and the Visualization of Mechanism-Based Inactivation. Angew. Chem. Int. Ed. Engl..

[22] Dai F., Jin F., Long Y., Jin X.L., Zhou B. (2018). A 1,8-naphthalimide-based turn-on fluorescent probe for imaging mitochondrial hydrogen peroxide in living cells. Free Radic Res..

[23] Li M., Fan J., Li H., Du J., Long S., Peng X. (2018). A ratiometric fluorescence probe for lysosomal polarity. Biomaterials.

[24] Lee D., Lim C.S., Ko G., Kim D., Cho M.K., Nam S.J., Kim H.M., Yoon J. (2018). A Two-Photon Fluorescent Probe for Imaging Endogenous ONOO^−^ near NMDA Receptors in Neuronal Cells and Hippocampal Tissues. Anal. Chem..

[25] Miao J., Huo Y., Liu Q., Li Z., Shi H., Shi Y., Guo W. (2016). A new class of fast-response and highly selective fluorescent probes for visualizing peroxynitrite in live cells, subcellular organelles, and kidney tissue of diabetic rats. Biomaterials.

[26] Palanisamy S., Wu P.Y., Wu S.C., Chen Y.J., Tzou S.C., Wang C.H., Chen C.Y., Wang Y.M. (2017). In vitro and in *vivo* imaging of peroxynitrite by a ratiometric boronate-based fluorescent probe. Biosens. Bioelectron..

[27] Qu W., Niu C., Zhang X., Chen W., Yu F., Liu H., Zhang X., Wang S. (2019). Construction of a novel far-red fluorescence light-up probe for visualizing intracellular peroxynitrite. Talanta.

[28] Yang D., Wang H.L., Sun Z.N., Chung N.W., Shen J.G. (2006). A highly selective fluorescent probe for the detection and imaging of peroxynitrite in living cells. J. Am. Chem. Soc..

[29] Wang C., Dong B., Kong X., Zhang N., Song W., Lin W. (2018). Dual site-controlled two-photon fluorescent probe for the imaging of lysosomal pH in living cells. Luminescence.

[30] Ren T.B., Xu W., Zhang Q.L., Zhang X.X., Wen S.Y., Yi H.B., Yuan L., Zhang X.B. (2018). Enhancing the Anti-Solvatochromic Two-Photon Fluorescence for Cirrhosis Imaging by Forming a Hydrogen-Bond Network. Angew. Chem. Int. Ed. Engl..

[31] Wang C., Dong B., Kong X., Zhang N., Song W., Lin W. (2018). A new xanthene-based two-photon fluorescent probe for the imaging of 1,4-dithiothreitol (DTT) in living cells. Luminescence.

[32] Czernel G., Matwijczuk A., Karcz D., Gorecki A., Niemczynowicz A., Szczes A., Gladyszewski G., Matwijczuk A., Gladyszewska B., Niewiadomy A. (2018). Spectroscopic Studies of Dual Fluorescence in 2-(4-Fluorophenylamino)-5-(2,4-dihydroxybenzeno)-1,3,4-thiadiazole: Effect of Molecular Aggregation in a Micellar System. Molecules.

[33] Murphy M.P., Smith R.A. (2007). Targeting antioxidants to mitochondria by conjugation to lipophilic cations. Annu. Rev. Pharmacol. Toxicol..

[34] Porteous C.M., Logan A., Evans C., Ledgerwood E.C., Menon D.K., Aigbirhio F., Smith R.A., Murphy M.P. (2010). Rapid uptake of lipophilic triphenylphosphonium cations by mitochondria in *vivo* following intravenous injection: Implications for mitochondria-specific therapies and probes. Biochim. Biophys. Acta.

[35] Kaur A., Brigden K.W., Cashman T.F., Fraser S.T., New E.J. (2015). Mitochondrially targeted redox probe reveals the variations in oxidative capacity of the haematopoietic cells. Org. Biomol. Chem..

[36] Saeki M., Kamisaki Y., Maeda S. (2000). Potentiation of carbachol-induced Ca2+ release by peroxynitrite in human neuroblastoma SH-SY5Y cells. Neurochem. Res..

[37] Pipicz M., Kocsis G.F., Sarvary-Arantes L., Bencsik P., Varga Z.V., Ferdinandy P., Csont T. (2017). Low-Dose Endotoxin Induces Late Preconditioning, Increases Peroxynitrite Formation, and Activates STAT3 in the Rat Heart. Molecules.

[38] Yoo B.K., Choi J.W., Shin C.Y., Jeon S.J., Park S.J., Cheong J.H., Han S.Y., Ryu J.R., Song M.R., Ko K.H. (2008). Activation of p38 MAPK induced peroxynitrite generation in LPS plus IFN-gamma-stimulated rat primary astrocytes via activation of iNOS and NADPH oxidase. Neurochem. Int..

[39] Asagiri K., Nakatsuka M., Konishi H., Noguchi S., Takata M., Habara T., Kudo T. (2003). Involvement of peroxynitrite in LPS-induced apoptosis of trophoblasts. J. Obstet. Gynaecol. Res..

[40] Takumida M., Popa R., Anniko M. (1998). Lipopolysaccharide-induced expression of reactive oxygen species and peroxynitrite in the guinea pig vestibular organ. ORL J. Otorhinolaryngol. Relat. Spec..

[41] Srikun D., Miller E.W., Domaille D.W., Chang C.J. (2008). An ICT-Based Approach to Ratiometric Fluorescence Imaging of Hydrogen Peroxide Produced in Living Cells. J. Am. Chem. Soc..

[42] Zhang J., Zhen X., Zeng J., Pu K. (2018). A Dual-Modal Molecular Probe for Near-infrared Fluorescence and Photoacoustic Imaging of Peroxynitrite. Anal. Chem..

[43] Hou J.-T., Yang J., Li K., Liao Y.-X., Yu K.-K., Yu X.-Q. (2014). A Highly Selective Water-Soluble Optical Probe for Endogenous Peroxynitrite. Chem. Comm..

[44] Kim J., Park J., Lee H., Choi Y., Ki Y. (2014). A Boronate-Based Fluorescent Probe for the Selective Detection of Cellular Peroxynitrite. Chem. Comm..

[45] Wang Z., Zhang F., Xiong J., Mao Z., Liu Z. (2020). Investigations of drug-induced liver injury by a peroxynitrite activatable two-photon fluorescence probe. Spectrochim. Acta Part A Mol. Biomol. Spectrosc..

[46] Xia L.L., Tong Y., Li L.S., Cui M.Y., Gu Y.Q., Wang P. (2019). A Selective Fluorescent Turn-On Probe for Imaging Peroxynitrite in Living Cells and Drug Damaged Liver Tissues. Talanta.

[47] Chen L., Cui M., Chen J., Xia L., Deng D., Gu Y., Wang P. (2020). A Novel Highly Selective Fluorescent Probe with New Chalcone Fluorophore for Monitoring and Imaging Endogenous Peroxynitrite in Living Cells and Drug-Damaged Liver Tissue. Talanta.

[48] Cui J., Zang S., Nie H., Shen T., Jing J., Zhang X. (2021). An Ict-Based Fluorescent Probe for Ratiometric Monitoring the Fluctuations of Peroxynitrite in Mitochondria. Sens. Actuators B Chem..

