# Supplementary material for: A Two-Photon Fluorescent Probe for the Visual Detection of Peroxynitrite in Living Cells and Zebrafish
Source: Molecules. 2022 Jul 29;27(15):4858. doi: 10.3390/molecules27154858 (PMC9369896; doi:10.3390/molecules27154858)
Supplement: Supplementary file 1 [file molecules-27-04858-s001.zip › molecules-1800475-supplementary.pdf]

molecules

Supplementary meterial

A Two-Photon Fluorescent Probe for the Visual Detection of Peroxynitrite in Living Cells and Zebrafish

Zhencai Xu [1,*], Jin Qian [2], Yufeng Ge [3], Yalan Wang [1] and Hongwei Chen [2,*]

[1] Guanyun People's Hospital, Lianyungang 222000, China; zhaoll0326@163.com
[2] Modern Education Technology Center, Department of Critical Care Medicine, The First Affiliated Hospital of Hainan Medical University, Hainan Medical University, Haikou 571199, China; jang9116@hainmc.edu.cn
[3] Department of Emergent Trauma Surgery, Qilu Hospital of Shandong University, Jinan 250012, China; geyufengqilu@163.com
* Correspondence: dingshun123@hainmc.edu.cn (Z.X.); chenhongwei@hainmc.edu.cn (H.C.)

1. Synthesis of HDBT-ONOO⁻

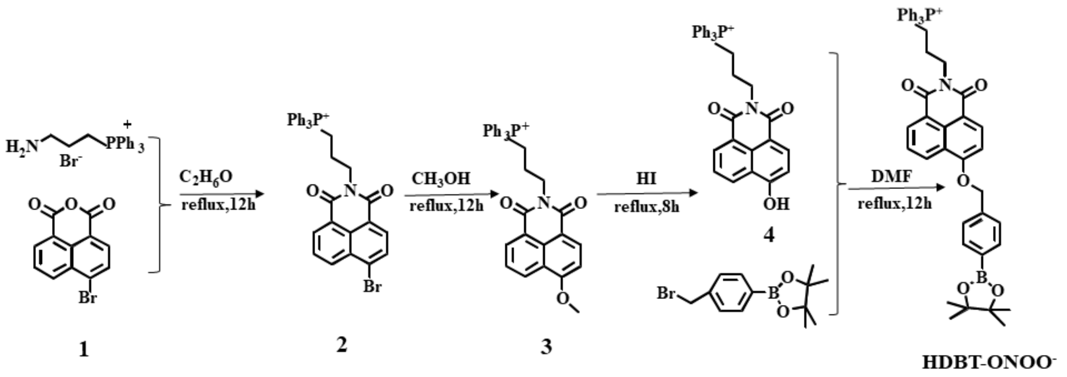

Figure S1. The synthetic route of HDBT-ONOO⁻.

Compound 2 Put compound 1 (277 mg, 10 mmol) and (3-aminopropyl) triphenylphosphonium (320 mg, 10 mmol) in a 100 mL round bottom flask containing 60 ml of absolute ethanol, add 2 ml of triethylamine, and heat reflux overnight. After the reaction is over, pour it into the water, adjust the pH to 2-3, solids appear, filter, and collect the solids. This compound was used directly in the next step.

Compound 3 The obtained compound 2 (2.5 mmol, 145 mg) was added to a round bottom flask containing 30 ml of methanol, potassium carbonate (20 mmol, 276mg) was added, and the mixture was heated to reflux overnight. After the reaction, it was poured into water, and a large amount of yellow solid appeared, filtered, dried, and used directly in the next step. ^{1}H NMR (600 MHz, $CDCl_3$) δ 8.57 (ddd, *J* = 5.6, 3.9, 1.2 Hz, 2H), 8.54 (d, *J* = 8.3 Hz, 1H), 7.78 – 7.66 (m, 6H), 7.59 – 7.30 (m, 10H), 7.05 (d, *J* = 8.3 Hz, 1H), 4.28 (t, *J* = 7.0 Hz, 2H), 4.14 (s, 3H), 2.46 – 2.40 (m, 2H), 2.11 – 2.05 (m, 2H). ^{13}C NMR (151 MHz, $CDCl_3$) δ 164.52, 163.92, 160.95, 133.74, 132.92, 132.27, 131.75, 130.85, 129.40, 128.90, 128.37, 125.96, 123.50, 122.19, 114.86, 105.25, 56.24, 40.58, 27.78, 27.30, 20.64.

Compound 4 The obtained compound 3 (2 mmol, 106 mg) was added to a round bottom flask containing 30 ml of hydroiodic acid and heated to reflux for about 8 hours. A large amount of yellow solid appeared, filtered, and washed with water 3 times. The obtained crude product was purified by column chromatography, dichloromethane:methanol=50:1-10:1, to obtain compound 4. ^{1}H NMR (600 MHz, DMSO) δ 11.89 (s, 1H), 8.53 (dd, *J* = 8.3, 1.1 Hz, 1H), 8.45 (dd, *J* = 7.3, 1.1 Hz, 1H), 8.34 (d, *J* = 8.2 Hz, 1H), 8.10 – 7.68 (m, 7H), 7.67 – 7.30 (m, 9H), 7.15 (d, *J* = 8.2 Hz, 1H), 4.13 – 4.09 (m, 2H), 2.50 –

2.45 (m, 2H), 1.78 (dd, J = 15.5, 7.6 Hz, 2H). ^{13}C NMR (151 MHz, DMSO) δ 164.27, 163.58, 160.72, 134.45, 134.06, 131.99, 131.63, 130.91, 129.17, 126.09, 110.39, 26.98, 20.590.

2. Main reagents and instruments

(3-aminopropyl) triphenylphosphonium, triethylamine, hydroiodic acid, absolute ethanol, and 4-Bromomethylphenylboronic acid pinacol ester were purchased from Aladdin. Commercial dyes and CCK-8 are from Thermo Fisher Scientific. Unless otherwise stated, all other chemicals are from commercial sources and are analytical reagent grade. All experiments always use ultrapure water.

Performed on silica gel plate TLC. Hitachi U-2910 for UV absorption spectroscopy experiments. Fluorescence spectroscopy experiments were performed on the Hitachi F4600 fluorescence spectrophotometer. 1H NMR and 13C NMR have obtained from BrukerAM 400 MHz and 100 MHz spectrometers, respectively. HR-MS data is obtained by Agilent 1290 infinity 6540 UHD accurate quality Q-TOF MS (Agilent, USA). The laser scanning confocal microscope (Olympus FV1000) uses a 60-fold objective lens to obtain cell fluorescence images. The images are collected and used Olympus FV10-ASW Ver.2.1 b software for processing. FBS, DMEM, and PBS were purchased from Gibco, USA. All kinds of cell lines were purchased from the Typical Culture Collection Committee of the Chinese Academy of Sciences (Shanghai, China). The probe (10 mg) was dissolved in DMSO (0.5 mL) and used for ^{1}H NMR, ^{13}C NMR, and HR-MS to characterize its structure.

3. Cell culture

HeLa, RAW 264.7, and HepG 2 cells were obtained from the Hainan Medical University, cultured in Dulbecco's modified Eagles medium (DMEM) with 10% newborn calf serum, and maintained at 37 °C in a humidified atmosphere containing 5% CO_2. The exponentially growing cells were used for all experiments.

4. Fluorescence image in cells

HeLa, RAW 264.7, and HepG2 cells were seeded into six-well plates at 1.5×10^5/well and incubated at 37 °C, 5% CO_2 for 24 hours. Cells were first incubated with HDBT-$ONOO^-$ (10 μM) for 30 min at 37°C, then washed 3 times with PBS (pH 7.4) to remove probes that were not absorbed into the cells.

5. Imaging of NO in zebrafish

For visualization of $ONOO^-$ in Zebrafish, the medium was replaced with a freshly prepared medium containing HDBT-$ONOO^-$ (10 μM), and the zebrafish was incubated within this fresh culture medium for 30 min. The zebrafish were then washed with E3 medium and fluorescence imaging was performed. For the positive control group, the above HDBT-$ONOO^-$ loaded zebrafish were then incubated with 1.2 mM SIN-1 ($ONOO^-$ donor) for another 90 min in an E3 culture medium. The zebrafish were washed with an E3 culture medium and then subjected to fluorescence imaging.

For visualization of endogenous $ONOO^-$ generation in zebrafish, the zebrafish were initially treated with LPS (2 μg/mL) for 2 h, and then incubated with HDBT-$ONOO^-$ (10 μM) for 30 min. The Zebrafish without LPS stimulation was employed as the control group. The zebrafish were washed with an E3 culture medium and then subjected to fluorescence imaging.

6. Fluorescence responses of HDBT-$ONOO^-$ to various interferents

Various stock solutions of metal ions were obtained by dissolving a series of nitrates (Na^+; Ca^{2+}; Mg^{2+}; Zn^{2+}; Fe^{2+}; Al^{3+}; Cu^{2+}) in deionized water. Various ROS were generated according to a reported procedure (ClO^-; NO; $^{\bullet}OH$; $\bullet O_2^-$; $^1O_2$; H_2O_2;) [41]. Peroxynitrite solution ($ONOO^-$) was synthesized according to the related literature report [25],

meanwhile, the probe HDBT-ONOO⁻ was dissolved in DMSO solution to prepare the stock solution.

7. Temporal response kinetics of HDBT-ONOO⁻ and ONOO⁻

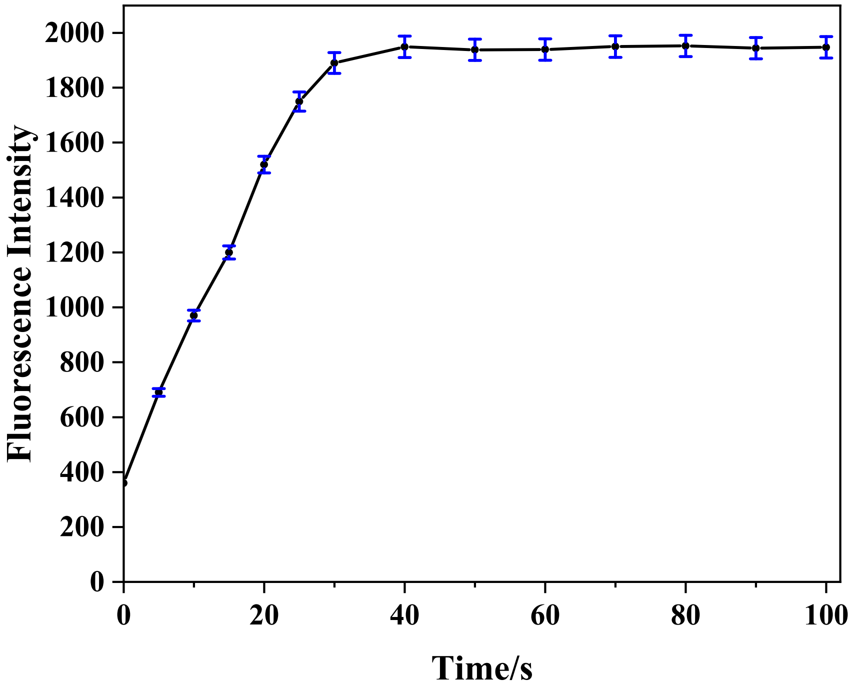

Figure S2. The fluorescence intensity of HDBT-ONOO⁻ (10 μM) varied with time in the presence of ONOO⁻ (20 μM) in PBS buffer (10 mM, pH 7.4). Repeat the experiment 3 times and express it as the average value (S.D.). λex = 450 nm.

8. Cytotoxic assay

HeLa, RAW 264.7, and HepG 2 cells were plated in DMEM with 10% FBS, cultured at 37°C in 5% CO_2 and 95% air. Three kinds of cells (8000 cells per well) were respectively seeded into 96-well plates and adherent culture for 24 h. Subsequently, the cells were incubated with 0, 10, 20, 30, 40, 50, 60, 70, and 80 μM (final concentration) probes (dissolved in DMSO) at 37°C in 5% CO_2 and 95% air at 24 h, under the same conditions as the control, untreated DMEM was also tested. Add CCK-8 solution (5.0 mg mL1, 10 mL) to each well. Then the plate was incubated in 5% CO_2 and 95% air for 1 h, and then the absorbance was measured at 450 nm using TECAN infinite M200pro.

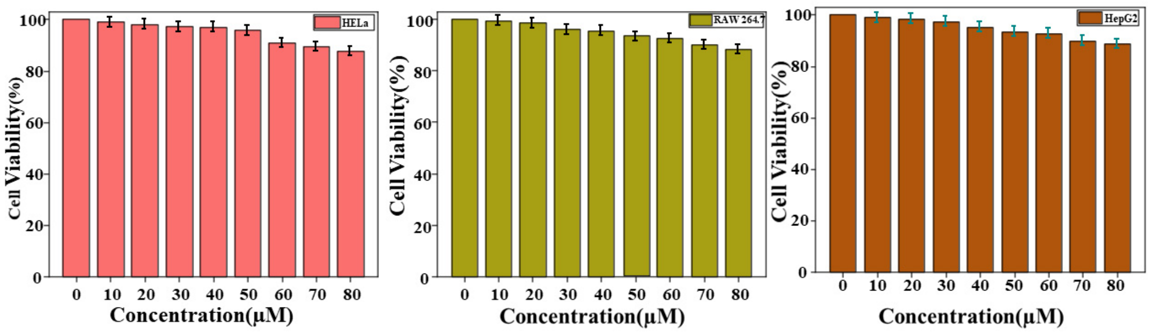

Figure S3. The cell viability of HDBT-ONOO⁻ against HeLa cells, RAW 264.7 cells, and HepG 2 cells. The experiments were repeated three times and the data were shown as mean (± S.D.). All data were obtained in PBS (10 mM, pH 7.4) at room temperature. λex = 450 nm.

9. Comparison of reported fluorescent probes and our proposed probe for $ONOO^-$.

Probe	LOD	Emission wave-length	Absorption wave-length	Detection time	Response type	Ref
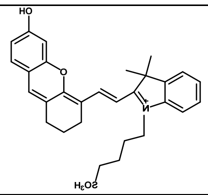	145 nM	712/680 nm	602 nm	3 min	Ratiometric	[42]
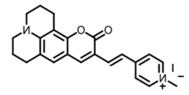	150 nM	652/493 nm	514 nm	4 min	Ratiometric	[43]
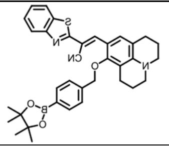	2.5 μM	540 nm	482 nm	30 min	Off-On	[44]
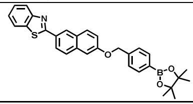	449 nM	508 nm	420 nm	15 min	Off-On	[45]
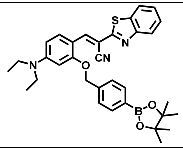	150 nM	530 nm	510 nm	20 min	Off-On	[46]
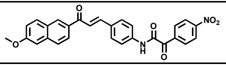	310 nM	560 nm	355 nm	15 min	Off-On	[47]
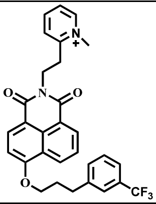	120 nM	558/454 nm	345 nm	20 min	Ratiometric	[48]
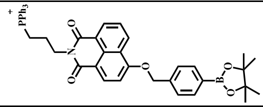	56 nM	558 nm	450 nm	30 s	Off-On	This work

10.1. H NMR, ^{13}C NMR, and HR-MS

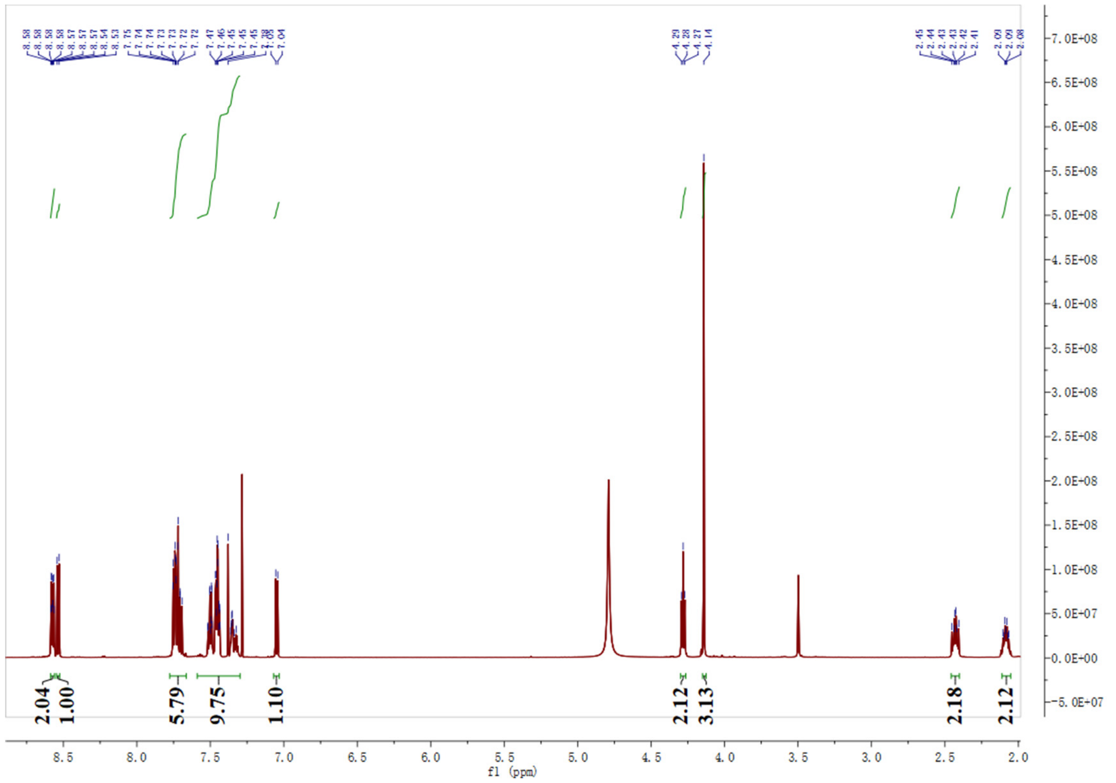

Figure S5. ^{1}H NMR of compound 3.

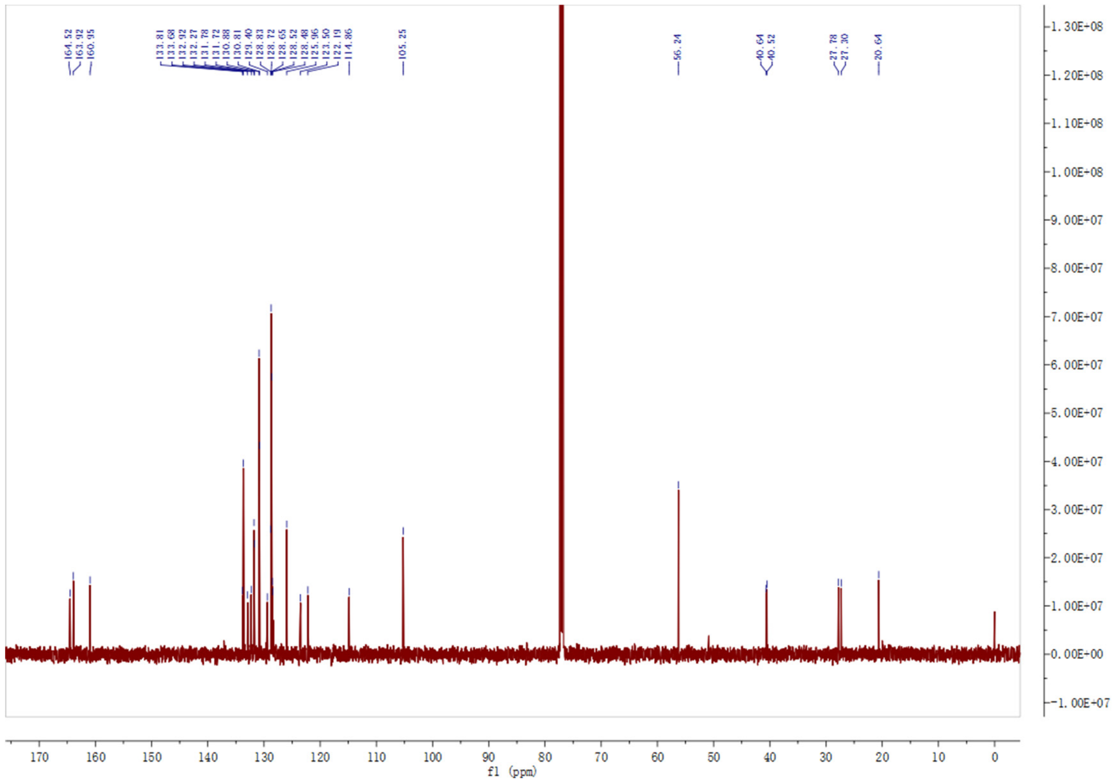

Figure S6. ^{13}C NMR of compound 3.

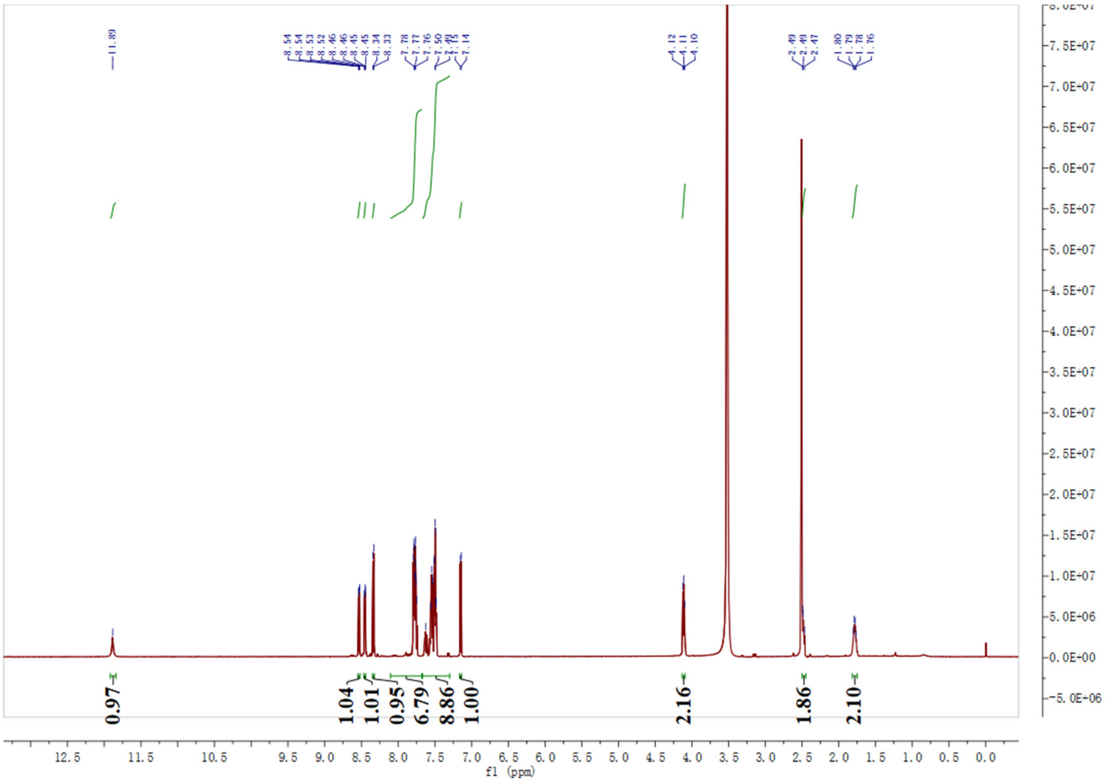

Figure S7. ^{1}H NMR of compound 4.

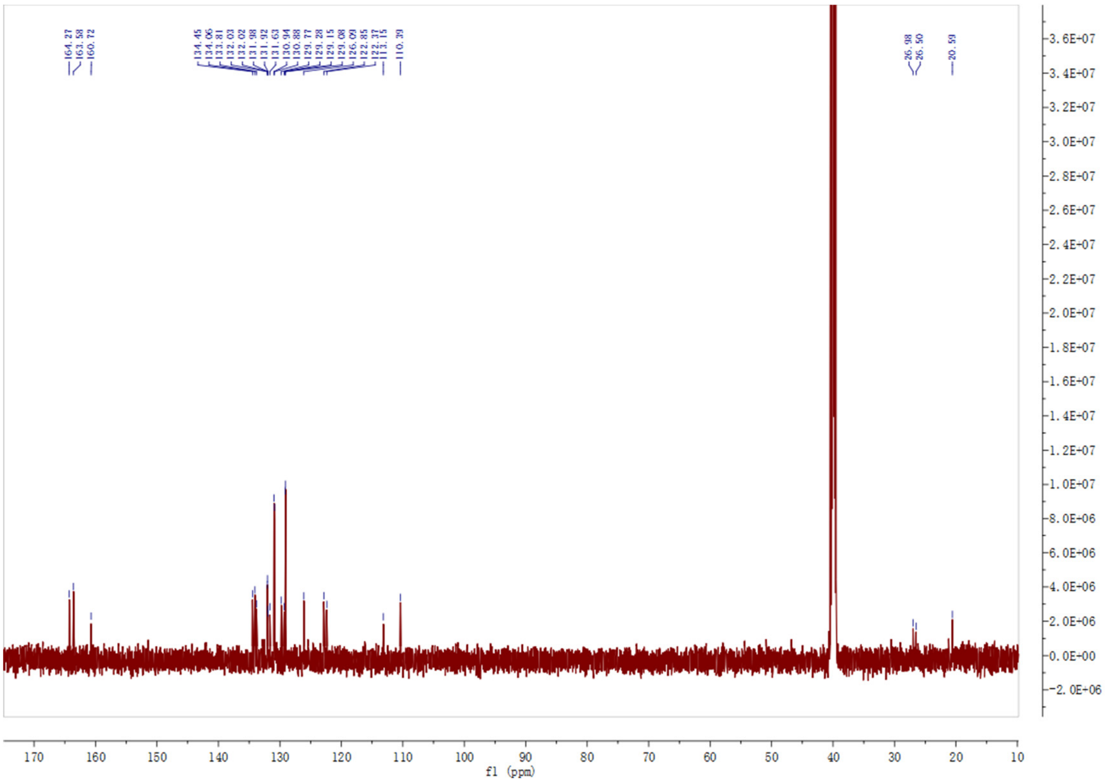

Figure S8. ^{13}C NMR of compound 4.

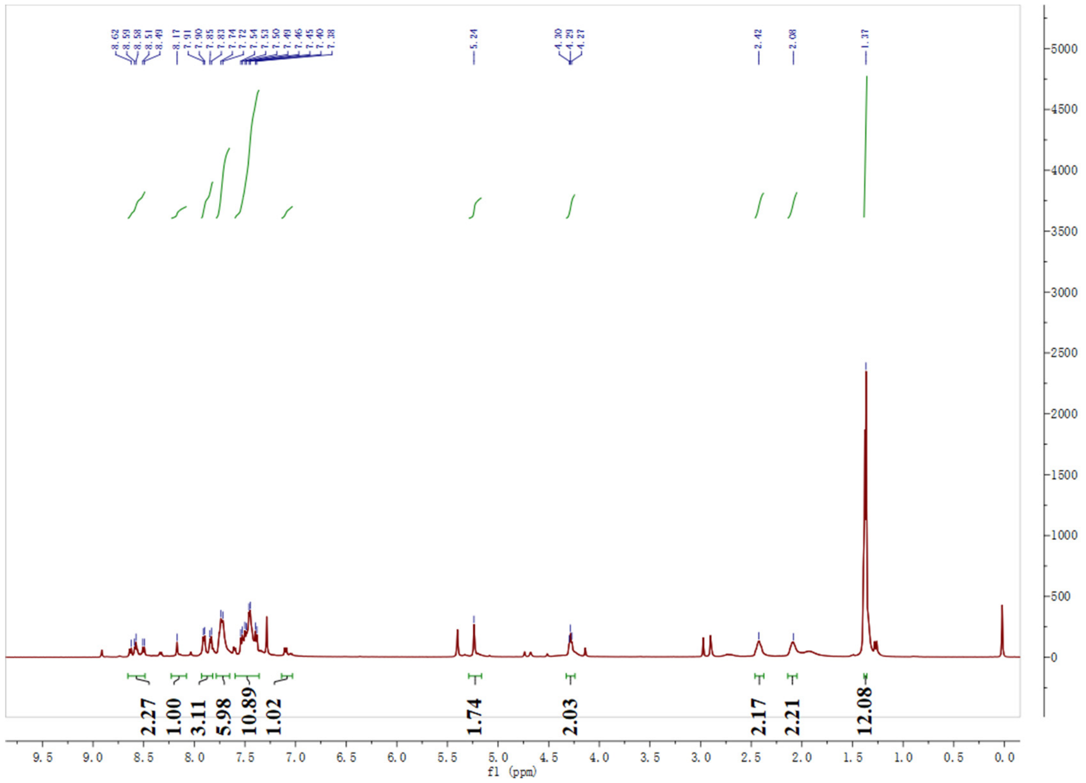

Figure S9. ^{1}H NMR of HDBT-ONOO⁻.

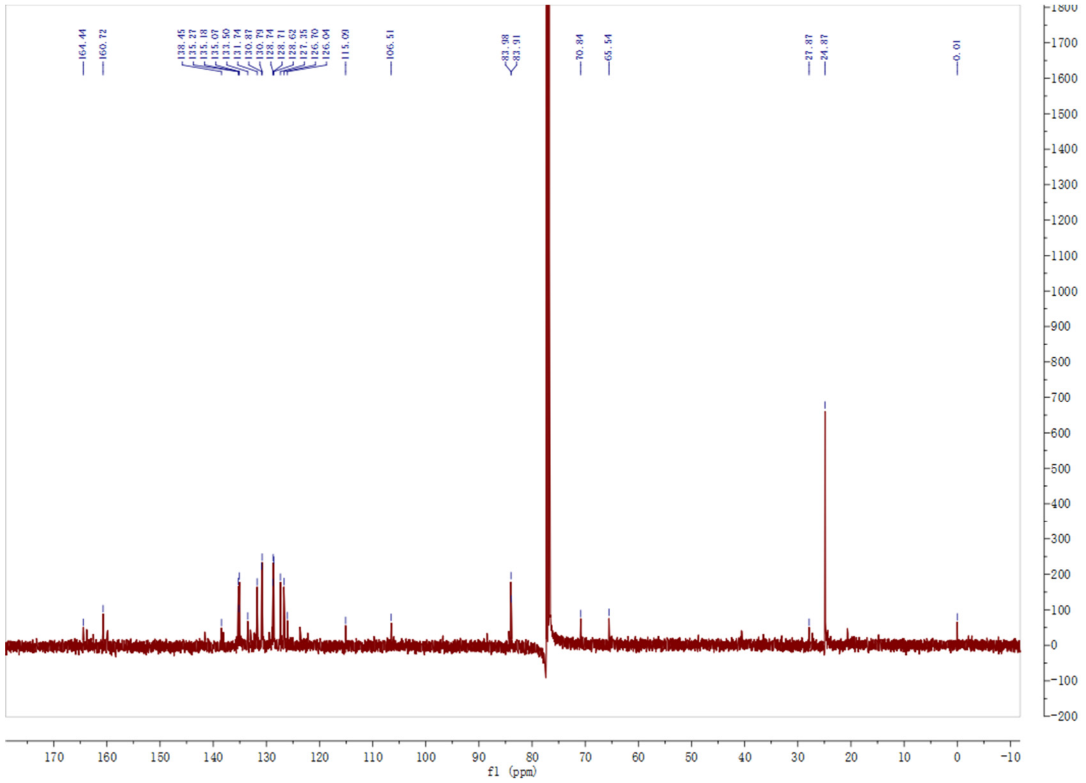

Figure S10. ^{13}C NMR of HDBT-ONOO⁻.

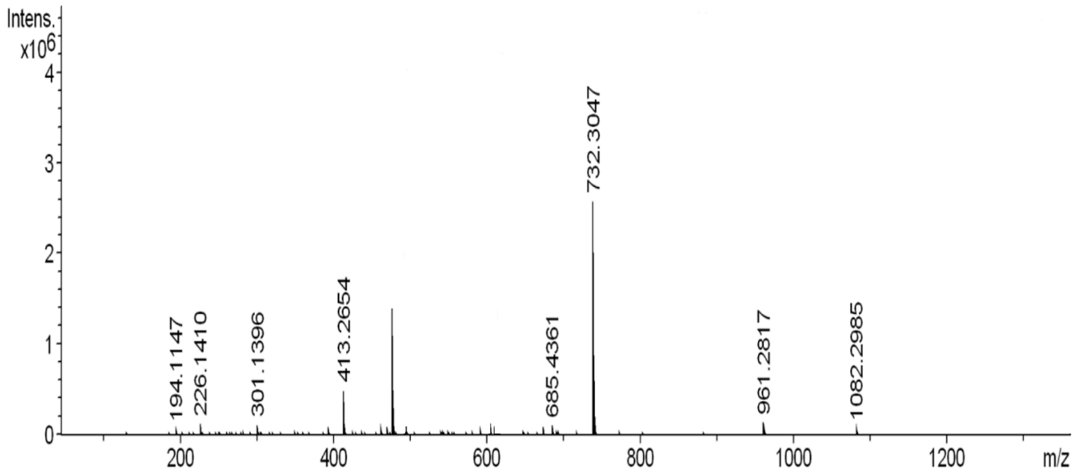

Figure S11. HR-MS of HDBT-ONOO⁻.
